# Inhibition Effect of Adipogenesis and Lipogenesis via Activation of *AMPK* in Preadipocytes Treated with *Canavalia gladiata* Extract

**DOI:** 10.3390/ijms24032108

**Published:** 2023-01-20

**Authors:** Ji Woo Hong, Ha Young Park, Han A. Kim, Yun Seon Hwang, Eun Jae Lee, Jin Woo Kim

**Affiliations:** 1 Department of Food Science, Sun Moon University, Natural Science 118, 70 Sunmoon-ro 221, Tangjeong-myeon, Asan-si 336-708, Chungnam, Republic of Korea; 2 Center for Next-Generation Semiconductor Technology, Sun Moon University, 70 Sunmoon-ro 221, Tangjeong-myeon, Asan-si 336-708, Chungnam, Republic of Korea; 3 FlexPro Biotechnology, Natural Science 128, 70 Sunmoon-ro 221, Tangjeong-myeon, Asan-si 336-708, Chungnam, Republic of Korea

**Keywords:** *Canavalia gladiate*, triglyceride metabolism, adipogenesis, lipogenesis, preadipocytes

## Abstract

The aim of this study was to investigate the effect of *Canavalia gladiata* extract (CGE) on the regulation of AMP-activated protein kinase (*AMPK*) in 3T3-L1 preadipocytes and evaluate the adipogenesis and lipogenesis mechanisms. In 3T3-L1 preadipocytes, lipid accumulation and differentiation were suppressed by 1.1, 1.3, and 1.4 times under the CGE treatment at 0.25, 0.5, and 1.0 mg/mL, respectively. The expression of the main genes involved in the inhibition of adipogenesis was evaluated at the mRNA level via a transcription-polymerase chain reaction. The extract at 1.0 mg/mL increased the mRNA expressions of AMPK and carnitine palmitoyl transferase-1 (*CPT-1*) by 1.9 and 1.2 times, respectively, while it decreased the expression of sterol regulatory element binding proteins-1c (*SREBP-1c*), peroxisome proliferator activated receptor-γ (*PPAR-γ*), CCAAT enhancer binding protein-α (*C/EBP-α*), and fatty acid synthase (*FAS*) by 1.1, 1.2, 1.8, and 1.5 times, respectively, indicating inhibition of the adipogenesis and lipogenesis potential of CGE. Gallic acid (4.02 mg/g) was identified as the main component of the CGE via LC-MS/MS and HPLC analysis. The results of this study suggested that CGE can be utilized as an anti-obesity food additive or medication by activating the AMPK-induced regulation and suppressing adipogenesis transcription factors.

## 1. Introduction

Body fat, which consists of triglycerides, sterols, and fatty acids, is essential for the maintenance of energy homeostasis and plays important roles such as supplying energy to the body, forming cell membranes, and absorbing fat-soluble vitamins [1]. In recent times, body fat hypertrophy and hyperplasia have occurred due to an imbalance in energy intake and consumption caused by the Westernized dietary habits associated with the rapid development of society and the economy, which resulted in obesity [2]. The production of excessive body fat causes metabolic syndromes, such as high blood pressure, diabetes, hyperlipidemia, and various other diseases that affect adults, including abnormalities in glucose metabolism [3]. Moreover, the critical cause of obesity is the enlargement and increase in the number of adipocytes during the differentiation of preadipocytes into adipocytes [4]. Research on the inhibition of this cell differentiation process is necessary to prevent and treat obesity. 

Preadipocyte differentiation is promoted by the stimulation of hormones such as 3-isobutylmethylxanthine (IBMX), dexamethasone (DEX), and insulin, as well as by the activation of preadipocyte-related genes and related transcription factors. The regulation of the latter is thus essential to prevent obesity [5]. The AMP-activated protein kinase (*AMPK*) is a regulator that plays a pivotal role in obesity prevention, activated by an increase in adenosine monophosphate (AMP) when energy in the cells is exhausted and adenosine triphosphate (ATP) is depleted [6]. The activated *AMPK* induces catabolism to maintain energy homeostasis and mediates the synthesis and decomposition of fatty acids, thus significantly influencing the metabolism of lipid and glucose [7]. The activation of AMPK inhibits lipid accumulation by suppressing sterol regulatory element binding proteins-1c (*SREBP-1c*), a main regulator of gene expression during preadipocyte differentiation [8]. The inhibition of *SREBP-1c* suppresses the expression of peroxisome proliferator activated receptor-γ (*PPAR-γ*) and CCAAT/enhancer binding protein-α (*C/EBP-α*), eventually limiting the formation of adipocytes [9]. Moreover, this process inhibits triglyceride accumulation by deactivating the ACC necessary for ATP consumption, arresting the production of malonyl-CoA, and then activating carnitine palmitoyl transferase-I (*CPT-1*) to promote the *β*-oxidation of fatty acids [10]. Thus, inhibiting triglyceride accumulation inside adipocytes through the suppression of preadipocyte differentiation is recognized as an important obesity-prevention strategy.

*Canavalia gladiata* is an annual perennial plant belonging to the *Apiaceae* family and is mainly cultivated in Sri Lanka and East Asia [11]. It contains several bioactive components, such as canavanine, gibberellin I, and gibberellin II, which are known to have antioxidant, anti-bacterial, and anti-diabetic properties [12,13]. Several bioactive molecules from natural products exert anti-obesity activity, inhibiting the adipogenesis of preadipocytes and adipose-derived stem cells. Recently, as *C. gladiata* has been reported to have excellent antifungal effects, the demand for it as a food and pharmaceutical material is increasing. In addition, its industrial use is increasing as in-depth studies on various bioactive functions of *C. gladiata* extract (CGE), including anti-obesity, anti-inflammatory, and anti-cancer functions, are being conducted. However, studies on the inhibition of preadipocyte differentiation and on the anti-obesity mechanisms of CGE have not been widely conducted. In the present study, this extract was confirmed to inhibit triglyceride accumulation and preadipocyte differentiation through the regulation of *AMPK*-inducing transcription factors. Furthermore, to elucidate the adipogenesis and lipogenesis mechanisms of CGE, the effects on the mRNA expressions of *AMPK*, *CPT-1*, *SREBP-1c*, *PPAR-γ*, *C/EBP-α*, and *FAS*, which are major genes involved in preadipocyte differentiation, were evaluated. The purpose of this study was to demonstrate that the CGE treatment significantly regulated both *AMPK* and *SREBP-1C* expressions, thereby affecting the expression of *CPT-1*, *PPAR-γ*, *C/EBP-α*, and *FAS*, sub-factors in the lipogenesis and adipogenesis pathways. The results obtained provide basic data that support the use of *C. gladiata* as an anti-obesity functional food and pharmaceutical product.

## 2. Results and Discussion

### 2.1. Effect of CGE on Cell Viability

**Figure 1:**
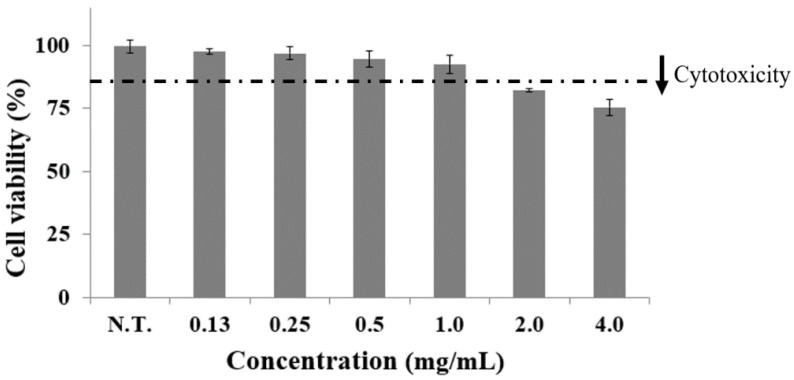
Effects of *C. gladiata* extract (0.0~4.0 mg/mL) on cell viability in preadipocytes. Bars represent the mean ± standard deviation of three independent experiments and asterisks indicate significant differences from the control.

Cell viability analysis was performed to evaluate the impact of the CGE on preadipocyte growth and to determine the extract concentration to be applied during subsequent experiments on the expression of adipogenesis and lipogenesis genes and on the inhibition of preadipocyte differentiation (Figure 1). When preadipocytes were treated with CGE concentrations of 0.0~4.0 mg/mL, a significant reduction in cell viability was specifically observed at 2.0 mg/mL or above, confirming that the extract affected this parameter. The cell viability of preadipocytes under treatment at 1.0 and 2.0 mg/mL was 92.8% and 82.8%, respectively. The CGE did not inhibit the growth of preadipocytes at ≤1.0 mg/mL, indicating that these concentrations did not inhibit cell viability. The above percentages were 4.64 times higher than that obtained in a previous study by Jeon et al., where a treatment with *Plantago asiatica* L. extracts at 1.0 mg/mL resulted in a cell viability of 20.0%, proving that the CGE is less cytotoxic to preadipocytes at this concentration compared to other existing natural products [14]. Therefore, 1.0 mg/mL was set as the maximum CGE concentration that would not inhibit preadipocyte growth during the subsequent experiments on the inhibition of preadipocyte differentiation and gene expression.

### 2.2. Effect of CGE on Lipid Accumulation

**Figure 2:**
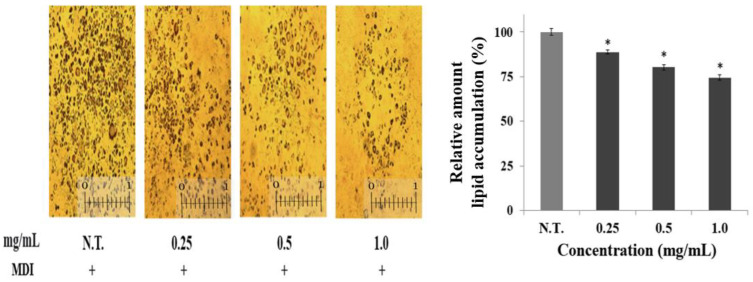
Effect of CGE on the lipid accumulation in adipocytes. Lipid droplets in adipocytes were fixed and stained with oil red O and were observed under a microscope of 64 magnification. Bar graphs represent the relative amounts of accumulated lipid based on untreated samples in adipocytes after being treated with CGE (0.0~1.0 mg/mL). Bars marked with asterisks are significantly different from the control (* *p* < 0.05).

Adipocytes store excess lipids or glucose as triglycerides and play a vital role in the regulation of lipid metabolism and energy homeostasis by supplying free fatty acids and glycerol when energy is needed [15]. Therefore, preadipocyte differentiation and increased lipid accumulation are closely connected to the development of obesity [16]. Thus, in the present study, this cell differentiation process was induced in order to evaluate the effect of the CGE on adipogenesis. The CGE was applied at concentrations of 0.25, 0.5, and 1.0 mg/mL during the treatment, and the accumulation of intracellular triglycerides was then compared by staining adipocytes (Figure 2). It was shown that the accumulation of triglycerides in these cells decreased in a concentration-dependent manner. Specifically, lipid accumulation decreased by 1.4 and 1.2 times with treatment of 0.5 and 1.0 mg/mL CGE, respectively, compared to the control group. This level of triglyceride reduction was higher than the 1.2-fold reduction reported for preadipocytes treated with turmeric extract at the same concentration, demonstrating that the CGE had a superior inhibitory effect on triglyceride accumulation compared to other natural products [17]. Therefore, it was confirmed that this extract inhibits the generation of lipids in adipocytes, thereby exerting an anti-obesity effect through reduced triglyceride accumulation. To determine the mechanism underlying this process, the expression of main genes centered on *AMPK* and involved in differentiation was then investigated.

### 2.3. Effect of CGE on Expression of Adipogenesis and Lipogenesis Genes

**Figure 3:**
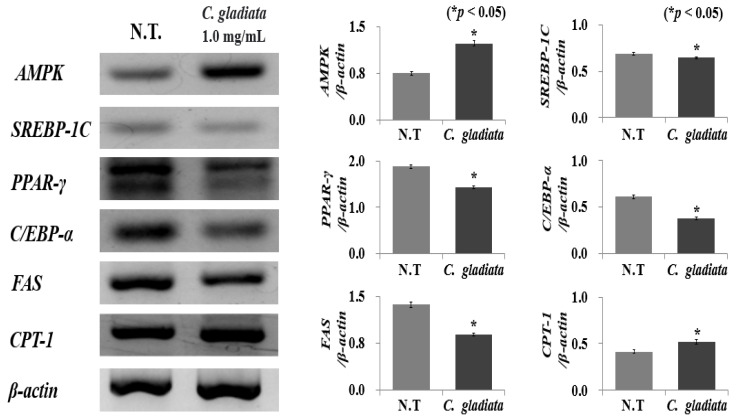
Effect of *C. gladiata* extract on *AMPK*, *CPT-1*, *SREBP-1c*, *PPAR-γ*, *C/EBP-α* and *FAS* mRNA expression levels in 3T3-L1. Values are expressed as mean ± standard deviation of three experiments. Bars marked with asterisks are significantly different from the control (* *p* < 0.05).

Cells use ATP and ADP as energy sources for metabolic processes and AMP generation. The increase in AMP activates AMPK based on the levels of depletion of intracellular energy, thus inducing catabolism to generate energy and allowing the maintenance of energy homeostasis [18]. In lipid metabolism, AMPK activation inhibits fatty acid synthesis and gluconeogenesis simultaneously [19]. Therefore, various anti-obesity mechanisms can activate AMPK during the treatment of this condition. To determine the inhibitory effect of *AMPK* activation on preadipocyte differentiation, the effect of the CGE on the expression of *AMPK*, *CPT-1*, *SREBP-1c*, *PPAR-γ*, *C/EBP-α*, and *FAS* mRNA (which are main factors in cell differentiation) was evaluated (Figure 3). The preadipocytes were treated with 1.0 mg/mL CGE, the maximum non-toxic concentration established in the previous experiment, and it was shown that the expression of *AMPK* and *CPT-1* increased by 1.9 and 1.2 times, respectively. On the other hand, the expression of *SREBP-1c*, *C/EBP-α*, *PPAR-γ*, and *FAS* decreased by 1.1, 1.2, 1.8, and 1.5 times, respectively. In particular, the expression of *AMPK* showed the highest increase, by 1.9 times, which exerted an anti-obesity effect by inhibiting lipogenesis in the body through the suppression of acetyl-CoA carboxylase and malonyl-CoA, the enzymes that induce fatty acid activity [20]. In addition, *AMPK* activated *CPT-1*, an enzyme that performs beta-oxidation by inhibiting malonyl-CoA, thus increasing the permeability of fatty acids in the mitochondria. This can be expected to exert an additional inhibition of adipogenesis and lipogenesis effect through the inhibition of triglyceride generation and promotion of fat oxidation [21]. 

In addition, the number of triglycerides accumulated following preadipocyte cell differentiation was determined by the increase or decrease in *SREBP-1c*, the upregulating mechanism of adipogenesis-related genes or signal transmitters [22]. *SREBP-1c*, which regulates the expression of genes involved in fatty acid metabolism and lipid biosynthesis in the early stages of preadipocyte differentiation, has been shown to inhibit the expression of the *PPAR-γ*, *C/EBP-α*, and *FAS* sub-factors to reduce lipid accumulation [23]. The CGE suppressed *PPAR-γ*, *C/EBP-α*, and *FAS* expression by inhibiting *SREBP-1c* as well as by increasing the expression of *AMPK* and *CPT-1*, which is expected to produce a high inhibition of adipogenesis and lipogenesis effect, making this extract a potentially successful candidate substance for the treatment of obesity. In this study, the effect of CGE on *AMPK* regulation in 3T3-L1 preadipocytes was investigated, and the adipogenesis and adipogenesis mechanisms were identified. Additionally, gene-level-based anti-obesity mechanism evaluation has limitations in evaluating protein expression and function; further in-depth studies, specifically through the use of in vivo anti-obesity models, are required to validate this and identify the anti-obesity mechanism.

### 2.4. Major Substance Analysis Using LC-MS/MS

**Figure 4:**
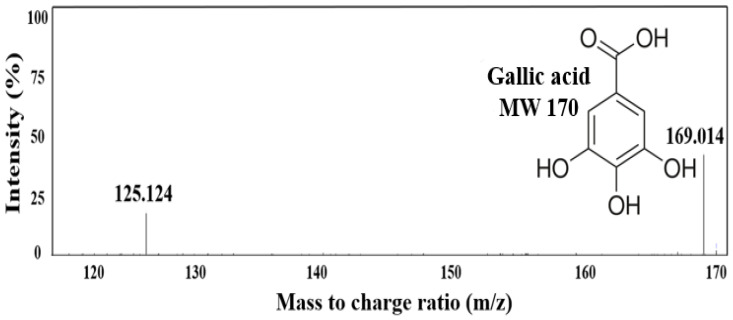
Mass Spectrum of LC-MS/MS fragmentation pattern of main substance, gallic acid (MW 170), from *C. gladiata* extract (full scan in negative ion mode).

In the previous analysis of the expression of adipogenesis and lipogenesis genes, the inhibition of triglyceride accumulation was confirmed by the increased expression of AMPK and CPT-1 and decreased expression of *SREBP-1c*, *C/EBP-α*, *PPAR-γ*, and *FAS*. Therefore, to explore the main substances contained in the CGE that inhibit preadipocyte differentiation, a qualitative analysis was performed using LC-MS/MS (Figure 4). A comparison of the molecular weight distribution of CGE in negative mode revealed the presence of two peaks, *m*/*z* 169.0 and 125.1. In this mode *m*/*z* was measured with one proton removed, and the substance detected at *m*/*z* 169.0 was predicted to be gallic acid (MW 170.0). This result was consistent with that reported by Gang et al., where LC-MS/MS analysis detected gallic acid as the main substance in the separation of major polyphenols in the CGE [24]. Subsequently, additional qualitative and quantitative analyses were conducted via HPLC to confirm the identity of the primary substance of the extract.

### 2.5. Quantification of Major Substance Using HPLC

**Figure 5:**
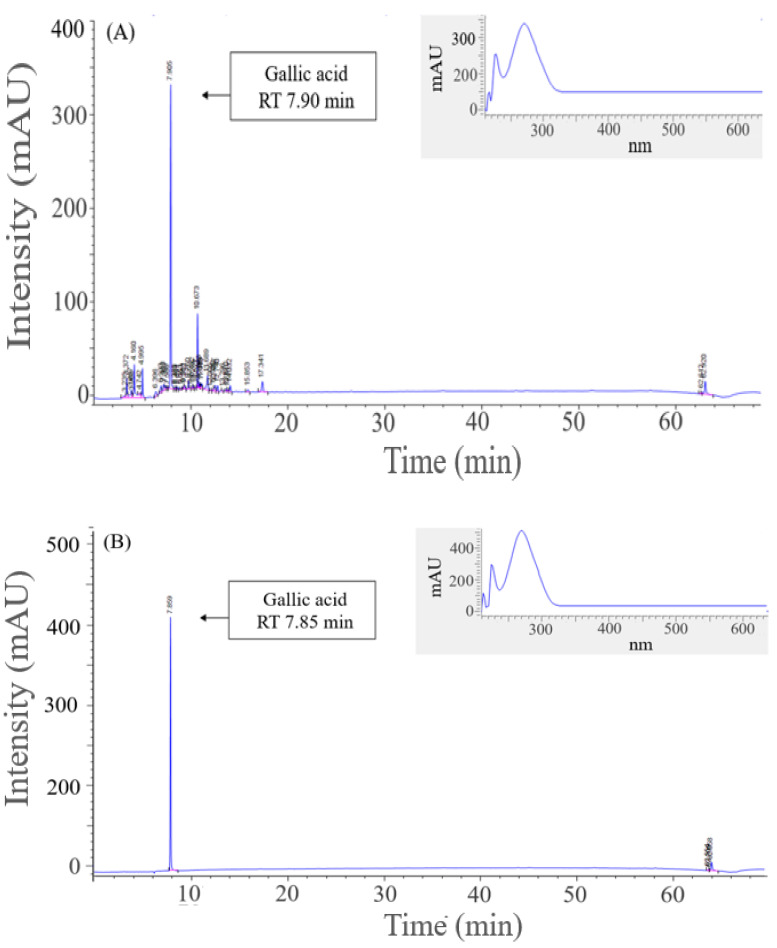
HPLC analysis for the quantitative analysis of main substances and polyphenols in *C. gladiata* extract. (**A**) chromatogram and DAD spectrum of *C. gladiata* extract, (**B**) chromatogram and DAD spectrum of gallic acid.

HPLC was used to perform qualitative and quantitative analyses based on retention time and DAD to validate the results of the earlier LC-MS/MS analysis. The RT of the major substances detected in the CGE was 7.9 min, which was highly consistent with that of 7.8 min found for gallic acid, the standard substance (Figure 5). The analysis of the corresponding peak with a DAD-based spectrum revealed a maximum absorption wavelength of 280 nm, which is consistent with that obtained when gallic acid was used as the standard substance in spectral form, reconfirming this compound as the main component of the CGE. The gallic acid concentration of 4.02 mg/g in the CGE was the highest among the peaks detected during HPLC analysis. Thus, this substance, which is a non-flavonoid tannin known for its anti-inflammatory, anti-cancer, liver protection, vascular disease prevention, and strong antioxidant functions, was identified as a major contributor to the inhibition of adipogenesis and lipogenesis effect of the CGE [25]. Furthermore, according to a study by Tan et al., gallic acid inhibited the expression of the main adipogenesis genes *SREBP-1c* and *PPAR-γ* by 37.2% and 51.1%, respectively, thus confirming its inhibition of adipogenesis effect through the inhibition of preadipocyte differentiation [26].

Catalase and peroxidase decompose the active oxygen generated in the body into water and oxygen molecules, thereby regulating homeostasis [27]. However, when an excessive production of active oxygen, lipid and protein denaturation, adipocyte metabolism disorders, and lowering of the body’s metabolic function are present, a homeostasis imbalance occurs, which ultimately leads to obesity. Gallic acid, the main substance of the CGE, is a typical polyphenol compound produced through the hydrolysis of tannin; it contains four hydroxyl groups and has a high active oxygen-scavenging activity, which is thought to contribute to increasing its anti-obesity effect [28]. According to previous studies by Song et al., *Sasa borealis* stem extracts containing gallic acid confirmed as the major substance exert anti-obesity effects on preadipocyte differentiation and lipid accumulation by regulating the expression of *CEBP-α* and *PPAR-γ*, which are early transcription factors involved in preadipocyte differentiation and attenuate hepatic steatosis in high-fat diet-induced obesity rats [29]. Overall, it was concluded that gallic acid identified as a major substance of CGE is highly effective as an anti-obesity substance, and it can also be used to remedy metabolic syndromes such as hyperlipidemia, high blood pressure, and arteriosclerosis.

## 3. Materials and Methods

### 3.1. Materials and Reagents

*C. gladiata* was purchased from Malgeundeul Co., Ltd. (Hongcheon, Korea) and kept frozen at −21 °C. Fetal bovine serum (FBS), new-born calf serum (NBCS), Dulbecco’s modified eagle medium (DMEM), and 0.05% trypsin-EDTA for cell culture were purchased from Thermo Fisher Sci., Inc. (Waltham, MA, USA). IBMX, DEX, insulin, and 3-(4,5-diimethylthiazole-2-yl)-2,5-diphenyl tetrazolium bromide (MTT) for preadipocyte differentiation and MTT assay were purchased from Sigma-Aldrich (St. Louis, MO, USA). Acetonitrile, acetic acid, and formic acid were purchased by high-performance liquid chromatography (HPLC) grade from Thermo Fisher Sci., Inc.

### 3.2. Preparation of CGE

*C. gladiata* was dried using a forced convection dry oven (FC 49, Lab House Co., Daejeon, Seoul, Korea) at 60 °C for 48 h and then pulverized into a particle size less than 0.42 mm using a grinder (HMF-3000S, Hanil, Buchoen, Korea). For the extraction of bioactive components from C. gladiata, 1.0 g of the dried sample was placed in a pressure-resistant Pyrex tube (PYREX-1636, Sci lab., Seoul, Korea) and mixed with 10.0 mL of 50% ethanol using a vortex mixer (VM-10, Daihan, Ltd., Wonju, Korea). Extraction was carried out at 60 °C for 30 min in the ultrasound extractor (250 W, SD-D250H, Daihan, Wonju, Korea). Then, the extract was subjected to solid–liquid separation at 10,000 rpm for 5 min using a centrifuge (LaboGene 1236R, Gyrozen Co., Daejeon, Korea).

### 3.3. Measurement of Preadipocyte Differentiation

Preadipocyte (3T3-L1) cells were obtained from the Korean Collection for Type Culture (KCTC, Jeongeup, Korea). 3T3-L1 preadipocyte with the inoculum density of 2.5 × 10^4^ cells/mL was cultured in DMEM supplemented with 10% NBCS, 1% penicillin, and MDI (0.5 mM IBMX, 1.0 mM DEX, and 1.0 μg/mL insulin) and cultured in a CO_2_ incubator (MCO-18AIC, Sanyo, Osaka, Japan) at 37 °C with 5.0% CO_2_. After 48 h, cells were fed with DMEM containing 10% FBS and 10 ug/mL insulin and cultured for an additional 48 h. Subsequently, the media was replaced with DMEM containing 10% FBS every 48 h.

### 3.4. Cell Viability Assay

The effect of CGE on the cell viability was determined using MTT assay. 3T3-L1 preadipocyte was seeded in a 96-well plate with 1 × 10^4^ cells/well of inoculum density, and different concentrations (0.0~4.0 mg/mL) of CGE were treated. After 48 h of cultivation, 0.1 mL MTT solution was added to induce formation of formazan at 37 °C and 0.1 mL DMSO was added after 4 h to dissolve formazan. Then, absorption was measured at 540 nm using a microplate reader (AMR-100, Allsheng, Seoul, Korea). In accordance with international standards (ISO 10993-5; Part 5: tests for in vitro cytotoxicity), the cytotoxic level was set below 85% cell viability [30]. The cell viability was calculated with the following equation:

(1) $$\mathrm{Cell}\;\mathrm{viability}\;\left(\%\right)=\left\{1\;-\;\frac{\mathrm{Abs}\;(\mathrm{sample})}{\mathrm{Abs}\;(\mathrm{control})}\right\}\;\times \;100\;$$

Sample: absorbance with CGE addition, control: absorbance with DMEM addition.

### 3.5. Lipid Accumulation Assay

Lipid accumulation of differentiated 3T3-L1 preadipocyte was measured by oil red O staining. The cells were washed with phosphate-buffered saline (PBS) and fixed with 10% formaldehyde solution for 30 min. Then, the cells were stained with oil red O solution for 60 min and washed with distilled water. The stained lipids were eluted with 99.5% isopropanol and measured using a UV–vis spectrophotometer at 497 nm by the following equation.

(2) $$\mathrm{Lipid}\;\mathrm{accumulation}\;\left(\%\right)=\left\{1\;-\;\frac{\mathrm{Abs}\;(\mathrm{sample})}{\mathrm{Abs}\;(\mathrm{control})}\right\}\;\times \;100\;$$

Sample: absorbance with CGE addition, control: absorbance with DMEM addition.

### 3.6. Measurement of Adipogenesis and Lipogenesis Genes Expression

RT-PCR was performed to determine the mRNA level of adipogenesis and lipogenesis genes including *AMPK*, *CPT-1*, *SREBP-1c*, *PPAR-γ*, *C/EBP-α* and *FAS* in 3T3-L1 preadipocyte. Cells were cultured in a T-25 cell culture flask (SPL Life Sci., Seoul, Korea) to a density of 3 × 10^5^ cells/mL. After collecting cells, total RNA was extracted using an AccuPrep^®^ universal RNA extraction kit and the expression levels were measured by spectrophotometer (NanoDrop™ 2000, Thermo Fisher, Waltham, MA, USA). Total RNA was reverse transcribed into cDNA using amfiRivert cDNA synthesis platinum master mix (GenDEPOT Co., Greenville, SC, USA). Then, cDNA was amplified with each primer (Cosmo Genetech, Seoul, Korea) *AMPK*, *CPT-1*, *SREBP-1c*, *PPAR-γ*, *C/EBP-α*, and *FAS* (Table 1). The initial denaturation was performed at 95 °C for 30 s, and the denaturation temperature was 59 °C for 30 s, annealing was at 72 °C for 30 s, and extension was at 72 °C for 7 min; samples were amplified for 35 cycles. Each PCR product was electrophoresed on 1.5% agarose gel and the intensity of bands visualized by using Gel Doc^TM^ XR + system (Bio-Rad Co., Richmond, CA, USA) to compare the band intensity with the housekeeping gene (*β*-actin).

### 3.7. LC-MS/MS Analysis

To conduct a qualitative analysis for determining the main component in CGE, phenolic components in CGE were analyzed by using liquid chromatography tandem mass spectrometry (LC-MS/MS) with a ROC C18 column. The column temperature and injection volume were set at 30 °C and 10 µL. The main components in CGE were analyzed by negative ion electrospray ionization using an electrospray ionization source and mass spectra were operated in the negative and positive ion mode between *m*/*z* 100 and 800 by a triple quadrupole mass spectrometer (TSQ Quantum Ultra EMR., Thermo Fisher, Waltham, MA, USA). The mobile phase consisted of two solvents, 0.1% formic acid in distilled water (A) and 0.1% formic acid in 50.0% acetonitrile (B), and a gradient elution system for the separation of polyphenols were as follows: 0~11 min, 95.0~0.0% A; 11~14 min, 0.0~0.0% A; 14~15 min, 0.0~95.0% A; and 15~20 min, 95.0~95.0% A.

### 3.8. HPLC Analysis

Quantitative and qualitative analyses of polyphenol in CGE were carried out using HPLC (Agilent 1260, Agilent Tech., Santa Clara, CA, USA) equipped with an autosampler, diode array detector (DAD), and Poroshell 120 EC-C18 column (4.6 × 150 mm, Agilent Technology, Santa Clara, CA, USA). The mobile phase was composed of two solvents, 99.9% acetonitrile (A) and 1.0% acetic acid (B), and gradient elution for the separation of polyphenols were as follows: 0~5 min, 0.0~15.0% A; 5~50 min, 15~50% A; 50~60 min, 50~100% A; and 60~64 min, 100~0.0% A. Main components in CGE were identified by comparing retention time (RT) as well as the DAD spectrum (190~640 nm) with polyphenol standards. A polyphenol standard including gallic acid with purity of 99.8% was purchased from Sigma-Aldrich.

### 3.9. Statistical Analysis

Data were expressed as the mean ± standard deviation (SD) for all experiments, and probabilities (*p*) of chance difference between groups were calculated according to Student’s *t*-test. A statistically significant test means that the test hypothesis is false or should be rejected and the criteria were set at *p* < 0.05.

## 4. Conclusions

This study aimed to validate the inhibition of anti-adipogenesis and lipogenesis effect of the CGE by evaluating the reduction in lipids by treatment with extracts and the inhibition of preadipocyte differentiation via *AMPK* activation. The results confirmed that triglyceride accumulation in adipocytes was reduced by 1.4 times when the extract at a concentration 1.0 mg/mL was added to preadipocytes. The evaluation of the mechanism underlying the CGE effects on lipogenesis and adipogenesis genes revealed an increase in the expression of *AMPK* and *CPT-1*, the main genes involved in preadipocyte differentiation, whereas the expressions of *SREBP-1c*, *PPAR-γ*, *C/EBP-α*, and *FAS* were reduced. The CGE as an inhibitor of triglyceride production was proved to have a high anti-obesity effect because it reduced the expression of subsequent regulators associated with *AMPK* expression by affecting the process of preadipocyte differentiation. This research indicated that CGE with inhibition of lipogenesis can be used as a natural adipogenesis agent, and it is expected to effectively inhibit the expression of *SREBP-1c*, *PPAR-γ*, *C/EBP-α*, and *FAS*, which are the major genes involved in the inhibition of lipogenesis and adipogenesis effect. In addition, LC-MS/MS and HPLC analysis confirmed that gallic acid was the main substance of the CGE and that, at a concentration of 4.02 mg/g, this compound inhibited the accumulation of triglycerides and affected preadipocyte cell differentiation. Therefore, the CGE is expected to have a high value for industrial application in health foods and pharmaceutical products.

## Figures and Tables

**Table 1:** Primer sequences used in reverse transcription-polymerase chain reaction (RT-PCR) of major genes related to adipogenesis and lipogenesis.

Genes	Forward (5’-3’)	Reverse (5’-3’)	Size (bp)
*AMPK* ^(1)^	CCATGCTGGAACTGATGGAG	CTGAACTGTGTGACCCAGCC	275
*CPT-1* ^(2)^	CGACATGCTCGGCCTCATAG	GCCAGAAGCCCCCAAGAAAC	813
*SREBP-1c* ^(3)^	GGTTTAGGGATGTTTGGGTTTT	AAGCCCACTTCATTTCATTGGT	122
*PPAR-γ* ^(4)^	AGCCGAGATAAAGCCAAACAAC	GAATCTCCTAGTCCTGGCTTGC	325
*C/EBP-α* ^(5)^	CCCTGAAATCCCAGCACTTC	GGCATGGCTGCTGTAGGGGT	137
*FAS* ^(6)^	CGACATGCTCGGCCTCATAG	GCCAGAAGCCCCCAAGAAAC	416
*β-actin*	AGCACAGAGCCTCGCCTTT	CTTAATGTCACGCACGATTTCC	295

^(1)^*AMPK*: AMP-activated protein kinase, ^(2)^*CPT-1*: carnitine palmitoyl transferase-1, ^(3)^*SREBP-1c*: sterol regulatory element binding proteins-1c, ^(4)^*PPAR-γ*: peroxisome proliferator activated receptor-γ ^(5)^ *C/EBP-α*: CCAAT/enhancer binding protein-α, ^(6)^ *FAS*: fatty acid synthase.

## Data Availability

No new data were created or analyzed in this study. Data sharing is not applicable to this article.

## Sample Availability

Samples of the compounds are not available from the authors.
